# DNA methylation-driven gene-based drug response prediction model for liver cancer: The critical role of GLS

**DOI:** 10.1371/journal.pone.0338091

**Published:** 2025-12-05

**Authors:** Shuang Zheng, Xi Yu, Xiaozhou Wang, Yi Ma, Yujia Han, Jing Zhang, Wei Wu, Chunyu Sun, Lijie He

**Affiliations:** 1 Department of Oncology, The People’s Hospital of Liaoning Province, People’s Hospital of China Medical University, Shenyang, Liaoning, China; 2 School of Pharmacy, China Medical University, Shenyang, Liaoning, China; 3 Department of Hepatobiliary Surgery, The Sixth People’s Hospital of Shenyang, Shenyang, Liaoning, China; Jamia Millia Islamia Central University: Jamia Millia Islamia, INDIA

## Abstract

**Background:**

Aberrant DNA methylation plays a pivotal role in cancer progression by enhancing oncogene activation or silencing tumor suppressor genes, contributing to malignant phenotypes. Methylation driver genes (MDGs) are characterized by an inverse correlation between DNA methylation levels and mRNA expression, making them critical targets for cancer research.

**Methods:**

We analyzed the liver hepatocellular carcinoma (LIHC) dataset from The Cancer Genome Atlas (TCGA) using the R package *MethylMix* to identify MDGs. Prognostic models were developed through univariate Cox regression, least absolute shrinkage and selection operator (LASSO) regression, and multivariate Cox regression to identify core genes. We further evaluated the associations of these genes with the tumor immune microenvironment, immune checkpoint inhibitors (ICIs), and chemotherapeutic sensitivity. Finally, liver cancer tissue organoid culture experiments combined with DNA methylation sequencing were conducted to validate predictions of drug sensitivity.

**Results:**

A total of 21 MDGs were identified, among which GNA14, glutaminase (GLS), and GNG4 were selected to construct a prognostic risk score model. The model demonstrated robust predictive performance, with Receiver Operating Characteristic (ROC) values of 0.723, 0.764, and 0.716 for 1-, 3-, and 5-year survival, respectively. Among these, GLS emerged as a key gene, showing low methylation levels and high mRNA expression, which were associated with poor prognosis, significant alterations in the tumor immune microenvironment, and differential sensitivity to ICIs and chemotherapeutic agents.

**Conclusion:**

The three-gene MDG-based prognostic model effectively predicts survival outcomes in LIHC patients. Moreover, the methylation status of GLS serves as a biomarker for assessing immune microenvironment characteristics, responsiveness to immunotherapy, and chemotherapy sensitivity, highlighting its potential as a therapeutic target in liver cancer.

## 1. Introduction

Hepatocellular carcinoma (LIHC) is one of the most prevalent malignancies worldwide, accounting for 4.7% of all cancer diagnoses and 8.3% of cancer-related deaths [1,2]. It primarily originates in intrahepatic bile duct epithelial or hepatocellular cells and predominantly affects males aged 30–50 [3]. Risk factors include alcohol consumption, smoking, obesity [4,5], type 2 diabetes [6,7], hepatitis B, and hepatitis C infections [8,9]. Despite advances in radiotherapy, chemotherapy, and targeted therapies, LIHC remains associated with poor prognosis due to late-stage diagnoses and low 5-year survival rates [5]. Identifying reliable molecular biomarkers for early detection and prognostic assessment is therefore crucial for improving outcomes.

DNA methylation (DNAm), a key epigenetic modification, plays a critical role in tumor initiation and progression by regulating gene expression. Aberrant DNAm patterns, such as hypomethylation-mediated gene activation and hypermethylation-induced gene silencing, have been identified across various cancers, including gastric [10], prostate [11], breast [12], esophageal, melanoma, colorectal, and lung cancers [13–15]. In LIHC, DNAm abnormalities contribute to cancer pathogenesis, influencing transcriptional regulation, immune evasion, and tumor progression [16–18]. Moreover, DNAm-based computational algorithms, such as those implemented in the R programming environment, have enabled the identification of methylation-driven genes (MDGs) with potential diagnostic and prognostic value [19]. Recent studies using DNAm probes have further highlighted the utility of methylation signatures in predictive modeling for LIHC outcomes [20]. However, a comprehensive analysis of MDGs and their clinical significance in LIHC remains insufficiently explored.

Recent studies have demonstrated the value of integrating DNA methylation and transcriptomic data to identify methylation-driven genes (MDGs) and elucidate their clinical relevance across multiple cancer types [21–23]. These investigations highlight the power of multi-omics bioinformatics approaches in uncovering key regulatory genes and constructing robust prognostic models. Building upon these methodological advances, the present study employed a similar integrative framework to systematically identify MDGs in liver hepatocellular carcinoma (LIHC) and to explore the potential of GLS as a clinically relevant biomarker and therapeutic target. Additionally, DNAm alterations have been implicated in modulating the tumor immune microenvironment (TME), a critical determinant of immunotherapy efficacy [24,25]. Leveraging methylation data from The Cancer Genome Atlas (TCGA), this study aims to construct a robust prognostic model for LIHC by identifying key MDGs associated with patient outcomes. By integrating bioinformatics approaches to analyze the relationships between TME, immune cell infiltration, immune checkpoint genes, and immunotherapy responsiveness, this study seeks to uncover actionable insights into the clinical application of MDGs. The findings have the potential to guide personalized treatment strategies, improve prognostic accuracy, and reduce recurrence risks in LIHC patients. This innovative approach underscores the translational value of bioinformatics-driven research in advancing LIHC management.

## 2. Materials and Methods

### 2.1. Methylation drive gene screening

From TCGA website (https://portal.gdc.cancer.gov/, on September 28, 2022) to obtain the LIHC transcriptome data sets and corresponding clinical information. In addition, the LIHC methylation dataset was obtained (https://bioconductor.org/packages/release/bioc/HTML/, on September 25, 2022). Validation criteria for methylation driver genes included assessment of the difference in gene mRNA expression between the normal population and the HCC group, assessment of DNA methylation levels in normal and tumor samples, and detection of the inverse correlation between DNA methylation level and mRNA expression of the same gene. Analysis was performed using the R packages “pheatmap”, “gplots”, “limma” and “MethylMix” with selection criteria set to FDR < 0.05, | log2FC | > 1 and corFilter > 0.2, following standard practices in the field [26,27] to ensure robust and biologically meaningful results.

### 2.2. Construction and assessment of a DMGs prognostic model

The LIHC group was divided into a training set (Trian) and a test set (Test) according to the ratio of 1:1. Subsequently, univariate Cox regression, absolute least shrinkage and selection operator (LASSO) regression and multivariate Cox regression analysis were performed on the Trian group samples to construct a risk scoring model. The score of the model formula is:


$$\begin{array}{c} \text{Prognostic risk score}=\sum_{\text{i}=\text{1}}^{\text{n}}\text{The corresponding coefficient}\times \text{ Expression of each gene} \end{array}$$

Trian patients were divided into low-risk and high-risk groups according to the median value. Kaplan-Meier (K-M) analysis and receiver operating characteristic (ROC) curve were used to evaluate the advantages and disadvantages of the model. Multivariate Cox regression and C-index were used to evaluate the independent prognostic characteristics of the model risk score using the selection criteria of P < 0.05, HR > 1 and 95% CI. Protein expression was confirmed using the Human Protein Atlas database. The R packages used are “survival”, “timeROC”and “climb”.

### 2.3. Identification and comprehensive analysis of core genes

GSVA enrichment analysis and Spearman correlation test were performed on risk scores and patterns to identify core genes. A nomogram was established to comprehensively evaluate multiple indicators. LIHC patients were divided into high and low groups according to the median of core genes, and stromal cells, immune cells, and TME were scored. Between-patient differences in the abundance of multiple immune cells were also assessed in the two study groups. It then forecasts the two groups of patients with sensitivity to the immune checkpoint inhibitors and 195 kinds of IC50 value concentrations of chemotherapeutic drugs. The R packages used are “limma,” “estimate,” “reshape2”, “ggpubr,” “preprocessCore,” “ggpub,” “ggExtra,” “vioplot,” “corrplot,” “ggplot2” and “protropic.”

### 2.4. Prospective study

#### 2.4.1. Sample collection and preliminary analysis.

We collected 5 cases of HCC patients who underwent surgery at the General Surgery Department of Liaoning Provincial People’s Hospital from March 29, 2023 to April 24, 2023. The study was conducted in accordance with the Declaration of Helsinki, and approved by the Ethics Committee of People’s Hospital of Liaoning Province (protocol code 2021HS011 and 18 August 2021 of approval). The inclusion criteria for the study are as follows:

(1) Age between 18 years and 75 years, gender not limited;
(2) ECOG score 0–1;
(3) Patients with primary liver cancer diagnosed clinically, including: imaging findings of liver nodules, when the liver nodules are 1–2 cm in size, at least 2 of the four imaging modalities (dynamic MRI/dynamic CT/Gd-EOB-DTPA-enhanced MRI (EOB-MRI)/contrast-enhanced ultrasound) must show typical features of liver cancer; when the liver nodules are > 2 cm in size, at least one of the four imaging modalities must show typical features of liver cancer;
(4) Patients judged clinically to require liver biopsy or resection for diagnosis and treatment;
(5) Study participants must have the ability to understand and sign an informed consent form, after being explained the study content, and voluntarily participate and sign an informed consent form before the start of the study-related procedures.

#### 2.4.2. Tumor tissue organoid culture was used to verify drug sensitivity.

We collected tumor tissues from five liver cancer patients who provided informed consent, for organoid culture and subsequent experiments. Tumor organoids were cultured in a medium containing 10% FBS, 20 ng/mL EGF, and 10 μM ROCK inhibitor at 37°C in a 5% CO_2_ incubator for 48 hours. Afterward, the drugs Temozolomide and Trametinib were added, and the organoids were further cultured for 48 hours, during which time their size and morphological changes were recorded.

### 2.5. Statistical analysis

All data were subjected to statistical analysis using R software. The normality of the data was assessed using the Shapiro-Wilk test. For normally distributed data, the mean±standard deviation (SD) was used, and intergroup comparisons were performed using the independent samples t-test. For non-normally distributed data, the median (interquartile range, IQR) was reported, and the Mann-Whitney U test was applied. Continuous variables were compared using either the independent samples t-test or the Mann-Whitney U test, depending on the distribution of the data, while categorical variables were compared using the chi-square test or Fisher’s exact test. Survival analysis was performed using the Kaplan-Meier method, and intergroup survival differences were compared using the Log-rank test. Cox proportional hazards regression was utilized to identify risk factors associated with clinical outcomes. The validity of the model was assessed using the C-index, and ROC curves were employed to evaluate the sensitivity and specificity of the model for prognosis. Decision Curve Analysis (DCA) was conducted to assess the clinical net benefit of the prognostic model across a range of threshold probabilities. Immune infiltration analyses were performed using multiple computational algorithms, including EPIC, TIMER, CIBERSORT, and xCell, to evaluate the associations between risk scores or gene expression and immune cell populations. Spearman correlation coefficient was used to assess the correlation between gene expression and clinical data. All statistical tests were two-sided, and a P-value of < 0.05 was considered statistically significant.

## 3. Results

### 3.1. Screening results of LIHC methylation driver genes

**Fig 1:**
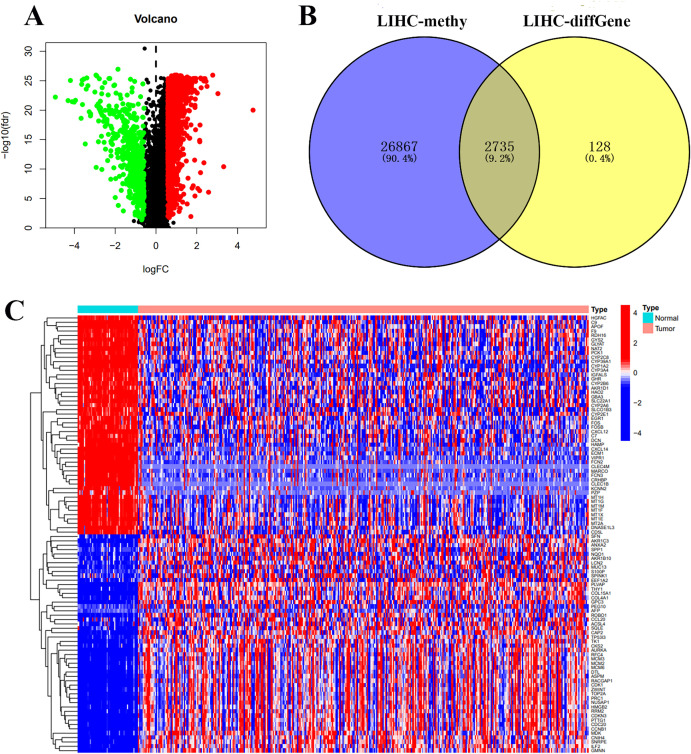
Analysis of differential methylation genes between LIHC patients and normal populations. (A) The volcano plot illustrates the differential methylation of driver genes between LIHC patients and normal controls, with red and green dots representing upregulated and downregulated genes, respectively. (B) The heatmap further displays the expression patterns of these genes across samples. (C) Statistical analysis of differential methylation genes reveals a set of genes significantly associated with methylation levels.

**Fig 2:**
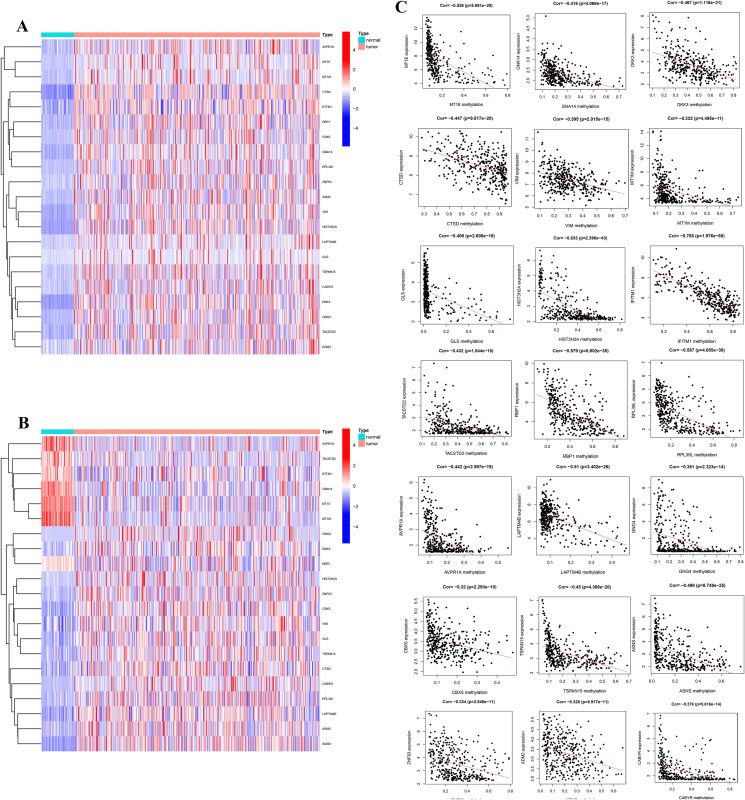
Relationship between methylation levels and expression of 21 specific genes. (A) and (B) Show heatmaps of the methylation levels and expression levels of methylation driver genes, highlighting a significant correlation between the methylation status of these genes and their expression. (C) Indicates a negative correlation between the methylation levels and expression levels of these 21 specific genes, suggesting that abnormal methylation of these genes may play a crucial role in the initiation and progression of LIHC.

To investigate the MDGs associated with the development and progression of LIHC, we performed differential expression analysis on the LIHC dataset from the TCGA database, which includes 371 LIHC patients. By integrating gene expression data with methylation levels, we identified a set of significantly altered MDGs. A total of 2,863 differentially related genes were identified, comprising 2,354 upregulated and 509 downregulated genes (Fig 1A and B). Among these, 2,735 genes were associated with methylation levels (Fig 1C). Additionally, 21 methylation driver genes were identified, including MT1E, GNA14, DKK3, CTSD, VIM, MT1M, glutaminase (GLS), HIST3H2A, IFITM1, TACSTD2, RBP1, RPL39L, AVPR1A, LAPTM4B, GNG4, CBX5, TSPAN15, ASNS, ZNF83, ADM2, and CABYR (Fig 2A and B). Fig 2C illustrates the relationship between the methylation levels and expression levels of these 21 genes, revealing a negative correlation where increased methylation corresponds to decreased expression. These findings suggest that methylation may play a pivotal role in regulating the expression of these genes, providing a foundation for constructing prognostic models and validating key genes in subsequent studies.

### 3.2. Methylation drive gene identification

**Fig 3:**
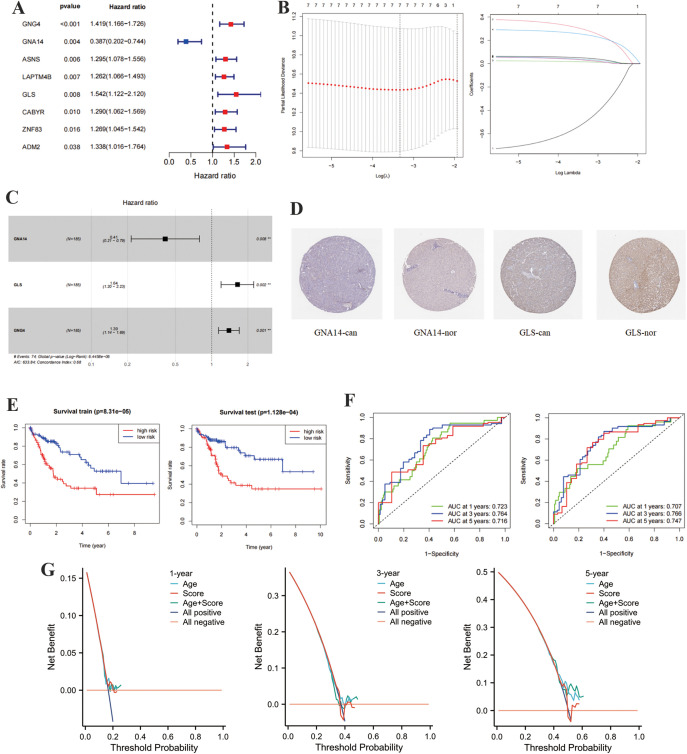
Prognostic modeling and validation of MDGs. (A) Univariate Cox regression analysis revealed the association between MDGs and the survival rate of LIHC patients, identifying eight genes significantly correlated with survival, namely GNA14, GLS, LAPTM4B, GNG4, ASNS, ZNF83, ADM2, and CABYR. (B) LASSO regression analysis, through cross-validation, selected the optimal λ value and further refined six prognostic-related core genes: GNA14, GLS, LAPTM4B, GNG4, ASNS, and ZNF83. (C) Multivariate Cox regression analysis confirmed the contribution of three key genes (GNA14, GLS, and GNG4) to survival prediction. (D) Representative immunohistochemical (IHC) images of GNA14 and GLS protein expression in normal liver and LIHC tissues obtained from the Human Protein Atlas (HPA) database. GNA14 expression was reduced in LIHC tissues, while GLS expression was increased. (E) Kaplan-Meier survival curves (Test group and Training group) demonstrated a significant difference in 5-year survival rates between high-risk and low-risk groups. (F) ROC curve analysis (TCGA and GEO databases) showed that the model’s risk prediction performance for 1-year, 3-year, and 5-year survival was 0.723, 0.764, and 0.716 (TCGA database) and 0.707, 0.766, and 0.747 (GEO database), respectively. These results indicate that the risk score model (GNA14, GLS, and GNG4) is reliable and adaptable, with strong predictive performance for prognostic evaluation in LIHC patients. (G) Decision Curve Analysis (DCA) for 1-, 3-, and 5-year overall survival (OS) in LIHC patients. The curves show that the risk score and the combined Age + Score model provide higher net clinical benefit than the “treat-all” or “treat-none” strategies across a wide range of threshold probabilities.


To further identify MDGs significantly associated with the prognosis of LIHC patients from the aforementioned key MDGs, we applied LASSO regression and multivariate Cox regression models to analyze the initially selected 21 core MDGs. Ultimately, three key genes (GNA14, GLS, and GNG4) were identified for the construction of a risk score model. This step aimed to discover core genes influencing patient survival, providing data support for subsequent studies. Using univariate Cox regression analysis and cross-validation, we identified eight genes (GNA14, GLS, LAPTM4B, GNG4, ASNS, ZNF83, ADM2, and CABYR) associated with prognosis in LIHC TCGA data (Fig 3A). These genes showed a significant correlation with prognosis and had the smallest absolute deviation value. LASSO regression analysis showed that GNA14, GLS, LAPTM4B, GNG4, ASNS, and ZNF83 have the lowest values and absolute deviation between CABYR, indicating a relationship with prognosis (Fig 3B). Multivariable Cox regression analysis confirmed that GNA14, GLS, and GNG4 are associated with prognosis in the risk score formula (Fig 3C). To further validate the expression patterns of key genes at the protein level, immunohistochemical (IHC) data were obtained from the Human Protein Atlas (HPA) database. The results revealed that GNA14 protein expression was lower in LIHC tissues (GNA14-can) than in normal liver tissues (GNA14-nor), while GLS expression was higher in LIHC tissues (GLS-can) compared with normal tissues (GLS-nor) (Fig 3D). These findings were consistent with the bioinformatics analyses, supporting that GNA14 may act as a protective factor, whereas GLS might serve as a risk factor in LIHC progression.

Risk score=−0.887* GNA14 expression + 0.492* GLS expression + 0.329*GNG4 expression

To validate the predictive power of the model, we assessed the clinical relevance of GNA14, GLS, and GNG4 for LIHC patients using survival analysis and ROC curves. It was found that patients were split into high and low-risk groups based on a median risk score of 0.1045, with the low-risk group having longer OS (Fig 3E). Similar results were seen in the Test group. ROC values were 0.723, 0.764, and 0.716 at 1, 3, and 5 years in the Train group, and 0.707, 0.766 in the Test group (Fig 3F). To further evaluate the clinical applicability of the prognostic model, Decision Curve Analysis (DCA) was conducted for 1-, 3-, and 5-year overall survival (OS) (Fig 3G). As shown in the DCA plots, the risk score model yielded a higher net benefit compared with the “treat-all” and “treat-none” strategies across a range of threshold probabilities, indicating favorable clinical usefulness. Moreover, the combined model integrating Age and Score (Age+Score) exhibited slightly improved performance compared with either factor alone, particularly at the 3- and 5-year time points. These results demonstrate that the prognostic model possesses potential value for individualized clinical decision-making in LIHC patients.

### 3.3. Independent prognostic analysis of risk scores and Nomogram analysis

**Fig 4:**
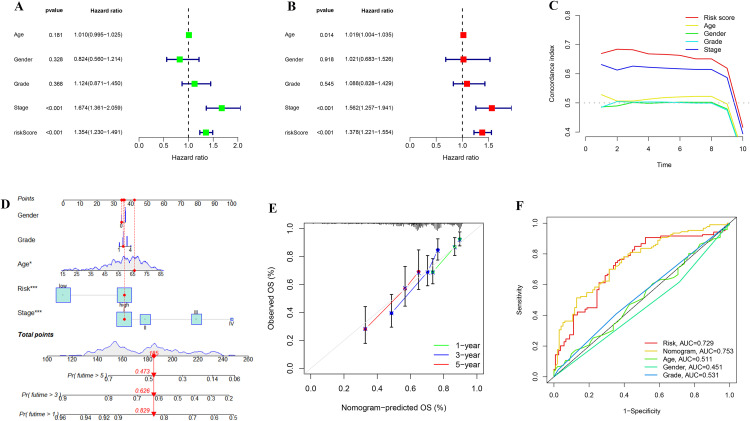
Independent prognostic analysis of risk scores and Nomogram analysis. (A) Univariate Cox regression analysis revealed that the risk score was significantly associated with overall survival (OS). (B) Multivariate Cox regression analysis further confirmed that the risk score is an independent prognostic factor. (C) The concordance index (C-index) curve demonstrated that the prognostic ability of the risk score outperformed other clinical features. (D) The nomogram integrates the risk score and clinical pathological features, providing a tool for individualized survival prediction. (E) Calibration curves showed good concordance between the predicted 1-year, 3-year, and 5-year survival rates and the actual observed values. (F) Multi- indicator ROC curve analysis proved that the nomogram’s predictive performance was superior to that of single indicators.

To validate the independent prognostic value of the risk score model, we performed univariate and multivariate Cox regression analyses on the clinical pathological features and risk scores of LIHC patients. Additionally, we constructed a nomogram to predict individual long-term survival, assessing the clinical applicability of the risk score model. Single factor Cox regression analysis indicated that pathological staging and risk score in LIHC patients have prognostic significance (Fig 4A). Multi-factor Cox regression analysis confirmed that these factors can independently predict patient prognosis (Fig 4B). The risk score had better predictive power than pathological staging, as shown in the C-index curve (Fig 4C). The nomogram (Fig 4D) depicted a patient’s clinicopathological characteristics and computed a cumulative risk score (N = 185), predicting 1-, 3-, and 5-year survival rates (1-year: 0.829; 3-year: 0.626; 5-year: 0.473). Fig 3e illustrated the stability of the calibration curve for the nomogram, while the area under the ROC curve demonstrated its predictive capacity over other clinicopathological features and risk scores (Fig 4F).

### 3.4. Identification of core genes

**Fig 5:**
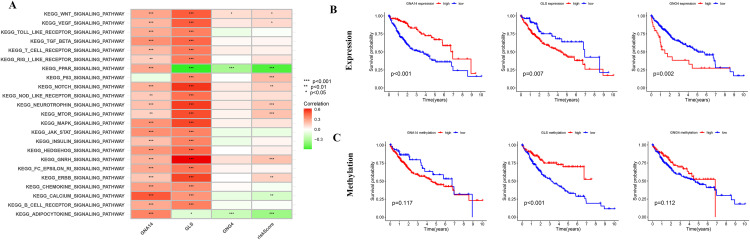
Identification analysis of core genes. (A) GSVA enrichment analysis revealed significant pathways associated with the core gene GLS, indicating its potential role in tumor metabolism and immune regulation. (B) Survival analysis demonstrated that high expression of the GLS gene was significantly correlated with poor prognosis in patients. (C) The methylation level of the GLS gene was also significantly associated with patient survival, further supporting its critical role in LIHC.

To further explore the biological significance of key genes in the risk score model, we conducted GSVA enrichment analysis and correlation analysis to identify the core gene GLS and investigated its expression levels in relation to patient prognosis. This analysis aimed to identify potential genes with crucial roles in the onset and progression of LIHC. According to the enrichment analysis results of Gene Set Variation Analysis (GSVA) presented in Fig 5A, pathways enriched by GLS exhibit a stronger correlation compared to other genes. The analysis of expression (Fig 5B) and methylation (Fig 5C) levels of model genes depicted reveals a significant association between GLS expression level, methylation level, and prognosis in patients with LIHC. In conclusion, GLS can be regarded as a pivotal gene.

### 3.5. The differences of TME, sensitivity to immunosuppressive agents and sensitivity to chemotherapy drugs in LIHC patients with differential expression of GLS

**Fig 6:**
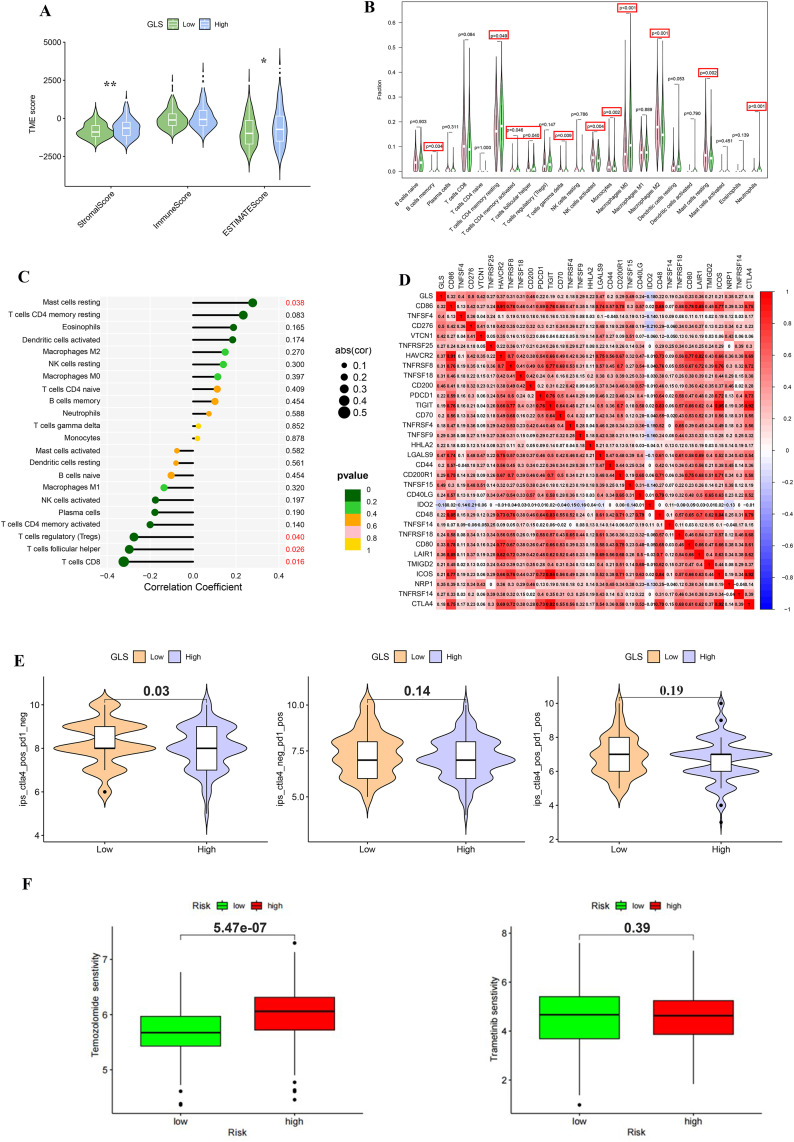
The differences in immune microenvironment, immunosuppressant sensitivity and chemotherapy drug sensitivity of LIHC patients with differential GLS expression were analyzed. (A) TME scores showed a significant increase in stromal scores in patients with high GLS expression, suggesting that GLS may regulate the tumor microenvironment. (B) Significant differences in the distribution of various immune cell types were observed between high and low GLS expression groups. (C) GLS expression was significantly correlated with specific immune cells, such as CD8^+^ T cells and regulatory T cells (Tregs). (D) The expression level of GLS was significantly positively correlated with immune checkpoint genes, including PD-1 and CTLA-4. (E) IPS immune microenvironment system scoring analysis revealed that, compared to PD-1 inhibitors, GLS-high expression patients showed better response to CTLA-4 inhibitors. (F) Sensitivity to Temozolomide differed significantly between high-risk and low-risk GLS expression groups, while no significant difference in sensitivity to Trametinib was observed, suggesting that GLS may serve as a predictive factor for Temozolomide sensitivity in LIHC patients.

To investigate the potential biological functions of the core gene GLS in LIHC patients, we analyzed the correlation between GLS expression levels and the tumor immune microenvironment (TME), immune checkpoint genes, and chemotherapy drug sensitivity. This analysis aimed to unveil the predictive value of GLS for immune therapy and chemotherapy response. As shown in Fig 6A, we found differences between StromalScore and ESTIMATEScore, but not ImmuneScore, among LIHC patient populations with different levels of GLS expression. But different types of immune cells in the two groups have significant differences in distribution (Fig 6B). Mast cells resting showed a positive correlation with GLS expression. GLS expression in LIHC patients’ tissues correlated with various immune cells, including B cells memory, T cells CD4 memory, T cells CD8, T cells follicular helper, T cells gamma delta, NK cells activated, Monocytes, Macrophages, Mast cells, and Neutrophils. Mast cells resting showed a positive correlation with GLS expression. There was a negative correlation between Tregs, T cells follicular helper, and T cells CD8 (Fig 6C). The expression level of GLS in LIHC patients’ tissues was strongly correlated with immune checkpoint-related genes (Fig 6D). Immune checkpoint inhibitors are important in treating LIHC patients. High GLS expression in LIHC tissues was significantly higher in the IPS CTLA4 positive + PD-1 negative group than in the group with low expression levels. IPS CTLA4 negative+PD-1 positive and IPS CTLA4 positive + PD-1 positive groups were not statistically significant (Fig 6E). The two groups of patients with differential GLS expression may have different sensitivities to 179 out of 195 chemotherapeutic drugs. One notable difference is in sensitivity to Temozolomide, with low-scoring patients being susceptible and high-scoring patients being less sensitive. Sensitivity to Trametinib showed no difference between the two groups (Fig 6F).

**Fig 7:**
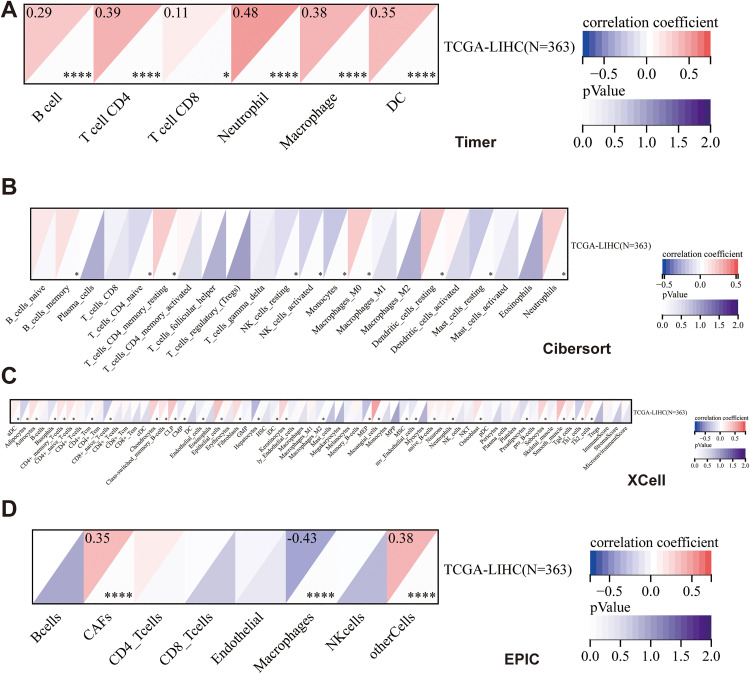
Immune infiltration analysis of the prognostic model in LIHC using multiple algorithms. (A) TIMER analysis showing correlations between risk score and immune cell types (B cells, CD4^+^ T cells, CD8^+^ T cells, neutrophils, macrophages, dendritic cells). (B) CIBERSORT-based estimation of 22 immune cell subsets in the TCGA-LIHC cohort. (C) xCell analysis displaying the distribution of stromal and immune signatures. (D) EPIC analysis showing positive correlations of the risk score with CAFs and macrophages, and negative correlations with CD8^+^ T cells.

To further explore the relationship between the prognostic model and the tumor immune microenvironment, multiple immune infiltration algorithms, including TIMER, CIBERSORT, xCell, and EPIC, were applied to the TCGA-LIHC cohort (Fig 7). As shown in TIMER analysis (Fig 7A), the risk score was positively correlated with the infiltration of B cell (*r* = 0.29, *p < *0.0001), T cell CD4 (*r* = 0.39, *p <* 0.0001), macrophages (*r* = 0.38, *p < *0.0001), neutrophils (*r* = 0.48, *p < *0.0001), and dendritic cells (*r* = 0.35, *p* < 0.0001), suggesting that patients in the high-risk group may exhibit an inflammatory and immunosuppressive tumor microenvironment. In CIBERSORT results (Fig 7B), several immune cell subsets, such as M0 and M2 macrophages, showed a positive association with the risk score, while resting memory CD4^+^ T cells tended to be negatively associated, indicating altered immune cell composition in high-risk patients. The xCell analysis (Fig 7C) further confirmed the enrichment of macrophage- and stromal-related signatures in the high-risk group, together with reduced levels of cytotoxic and helper T cell subsets. Similarly, EPIC analysis (Fig 7D) revealed that the risk score was positively correlated with CAFs (*r* = 0.35, *p < *0.0001) and other cells (*r* = 0.38, *p < *0.0001), but negatively correlated with macrophages (*r* = –0.43, *p <* 0.0001), further supporting that immune suppression and stromal activation are prominent features in high-risk LIHC patients.Collectively, these findings indicate that the prognostic model is closely related to immune cell infiltration characteristics, suggesting that the high-risk group may exhibit a more immunosuppressive tumor microenvironment.

### 3.6. Validation of predictive accuracy for chemotherapeutic drug sensitivity through tumor organoid drug sensitivity assay

**Fig 8:**
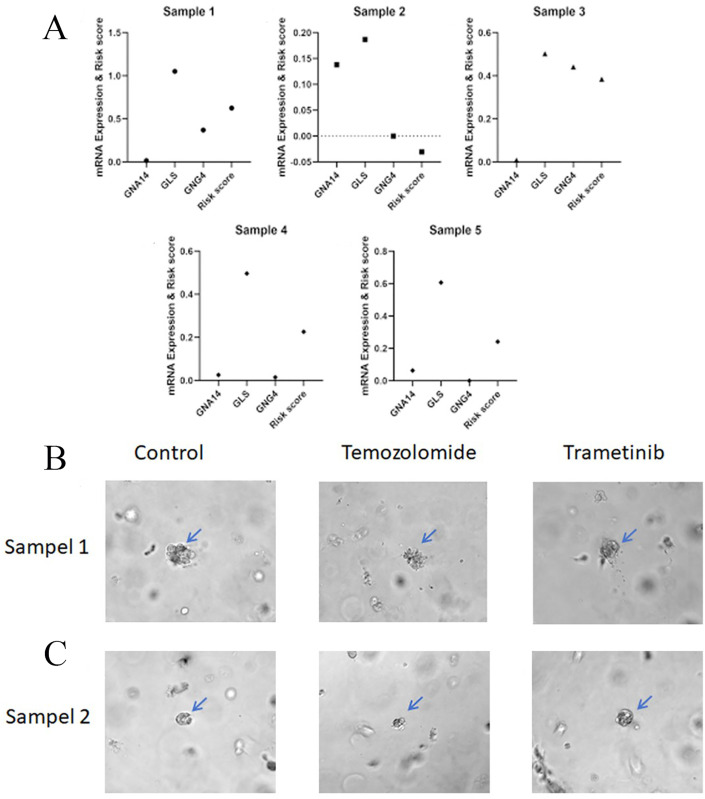
The results of the prediction of chemotherapeutic drug sensitivity of the samples and the drug sensitivity test of tumor organoids were analyzed. (A) Expression levels of GLS, GNA14, and GNG4, along with model risk scores, were measured in five clinical liver cancer samples. (B) Sensitivity testing of liver cancer organoids (Sample 1) to Temozolomide and Trametinib showed no significant differences in organoid size and morphology between drug-treated and control groups. (C) In contrast, liver cancer organoids from Sample 2 showed significant shrinkage after Temozolomide treatment, indicating higher sensitivity to this drug. The results were consistent with the model’s predictions, further confirming the accuracy of the model in predicting chemotherapy drug sensitivity.

To validate the predictive accuracy of the model for chemotherapy drug sensitivity, we employed tumor organoid culture technology to assess the response of LIHC samples to different chemotherapy drugs. This experiment aimed to provide in vitro evidence supporting the application of GLS in predicting chemotherapy drug sensitivity. As shown in Fig 8A, among the five LIHC samples, sample 1 had the highest GLS expression and risk score, while sample 2 had the lowest GLS expression and risk score. Therefore, it was predicted that sample 1 had no difference in sensitivity to Temozolomide and Trametinib, and sample 2 had high sensitivity to Temozolomide and low sensitivity to Trametinib. Fig 8B illustrates that the liver cancer organoids of Sample 1 exhibited similar size and morphology to the control group following treatment with Temozolomide and Trametinib, indicating comparable sensitivity to both agents. In contrast, the liver cancer organoids of Sample 2 showed significantly reduced size and marked morphological changes after Temozolomide treatment compared to the control group and the Trametinib treatment group, suggesting a potential differential sensitivity between Temozolomide and Trametinib in this sample (Fig 8C). These findings align with our predicted organoid drug sensitivity experiment results for Sample 1 and Sample 2.

## 4. Discussion

LIHC remains a prevalent malignancy despite advancements in early detection techniques and treatment strategies, such as local resection and liver transplantation [28]. However, the long-term prognosis for LIHC patients is poor, presenting a significant challenge for healthcare providers. While conventional prognostic factors- such as liver function, vascular invasion, tumor stage, and biomarkers-are commonly used to guide treatment decisions, their predictive accuracy is limited due to the considerable heterogeneity of LIHC patients [29–31]. Among traditional biomarkers, alpha-fetoprotein (AFP) is a reliable predictor of tumor recurrence, yet its predictive scope remains inadequate [32]. This highlights the necessity of integrating multi-omics data to identify novel prognostic biomarkers, which may enhance the precision and clinical utility of prognostic models for LIHC.

Epigenetic modifications, particularly DNAm, play a critical role in the pathogenesis and progression of cancer [33]. Advances in microarray and sequencing technologies have facilitated the identification of aberrant DNAm patterns, including hypermethylation of promoter CpG islands, which can silence tumor-suppressor genes and activate oncogenes, contributing to tumor initiation and progression [34]. In LIHC, aberrant DNAm has been linked to tumor aggressiveness and poor prognosis [35,36]. This study sought to explore MDGs in LIHC through bioinformatics analysis and developed a robust prognostic risk model validated for clinical relevance using ROC curve analysis.

Numerous studies have documented epigenetic alterations in patients with LIHC and have identified potential prognostic biomarkers. For instance, Li et al. investigated the association between MDGs and LIHC, revealing that six of these markers were significantly linked to LIHC prognosis [37]. Guorun Fan and colleagues investigated the association between MDGs and LIHC by identifying core genes through PPI interaction networks and conducting correlation analyses, rather than constructing a model [38]. Our study used various regression analyses to investigate the relationship between MDGs and prognosis in LIHC patients. We also developed a prognostic risk score model that was validated for accuracy through ROC curve analysis.

Furthermore, the core gene GLS, a subtype of glutaminase (GA) in human cells and a mitochondrial enzyme responsible for catalyzing the initial step of glutamine metabolism, has been identified [39]. Literature suggests that oncogenes influence GLS and contribute to the proliferation of malignant tumor cells across various cancers. Additionally [40], GLS has the ability to modulate the antioxidant defense mechanism within cells by elevating glutathione (GSH) levels and decreasing reactive oxygen species (ROS) levels, ultimately safeguarding cells against oxidative stress [41]. Research shows a link between abnormal GA metabolism and liver disease characteristics, with the gene GLS playing a role in liver disease progression in individuals with cirrhosis [42]. Studies have demonstrated the involvement of GLS in the migration and invasion of LIHC cells [43]. Multiple studies have shown that the oncogene Myc can increase glutamine synthetase expression through promoter demethylation, promoting glutamine metabolism and potentially influencing tumor growth driven by Myc [44,45]. Consequently, our research involved a comprehensive examination of GLS, revealing that it functions as a key gene in methylation regulation and is prominently expressed and hypermethylated in LIHC. The hypermethylation level of gene promoter regions is negatively correlated with gene expression. Furthermore, our study revealed a significant correlation between the expression of GLS in the tissues of patients with LIHC and TME as well as various immune cells. We identified a correlation between the expression of GLS and immune checkpoint genes, such as PDCD1, TNFSF15, CD80, and CTLA4, providing predictive value for the efficacy of CTLA4. Finally, our prospective study showed that GLS expression level is one of the predictors of chemosensitivity in LIHC patients.

Besides GLS, two additional genes incorporated into the prognostic model- GNA14 and GNG4-may also contribute to hepatocellular carcinoma biology. GNA14, encoding a Gα subunit of the G-protein complex, participates in GPCR signaling and has been linked to angiogenesis, vascular remodeling, and tumor progression in several cancers, including HCC [46]. GNG4, which encodes a Gγ subunit, has been implicated in regulating PI3K/AKT and MAPK pathways and may influence tumor immune regulation and metabolic adaptation [47]. Although detailed mechanistic validation was beyond the scope of this study, their inclusion in the model aligns with known oncogenic signaling pathways and highlights their potential prognostic and therapeutic relevance.

Although our analysis identified methylation-driven genes using MethylMix by integrating DNA methylation and gene expression data at the gene level, this approach does not explicitly distinguish between promoter and gene body methylation, which may exert opposing regulatory effects. Future studies will therefore conduct region-specific analyses incorporating CpG island context (shore, shelf, and open sea) to provide a more detailed mechanistic understanding of the methylation patterns underlying GLS regulation and other candidate genes. While MethylMix was adopted in this study for its robustness and biological interpretability, alternative algorithms such as ELMER and MethSig employ distinct modeling strategies and could yield complementary insights. Integrating results across multiple computational frameworks may facilitate cross-validation and improve the identification of robust methylation-driven biomarkers in liver cancer. In addition, recent advances in artificial intelligence (AI) and deep learning–based frameworks have opened new avenues for drug repurposing, particularly through the integration of multi-omics and pharmacogenomic data to uncover novel therapeutic opportunities [48]. For instance, AI-driven models have been applied to systematically predict compound–disease associations and prioritize repurposed agents with potential efficacy in specific tumor contexts [49]. These approaches underscore the growing potential of computational drug repositioning in precision oncology. Although the present study primarily utilized pharmacogenomic correlations to identify candidate drugs associated with GLS-related pathways, incorporating AI-based repurposing frameworks in future work could further enhance the translational relevance and predictive precision of our prognostic model. Furthermore, given that TCGA-LIHC does not include patients treated with ICIs, the predictive findings regarding GLS and immune checkpoint gene associations should be interpreted with caution. Future validation using ICI-treated cohorts, such as IMvigor210, will be essential to confirm the clinical applicability of these immunogenomic correlations once suitable datasets become available.

In addition to conventional pharmacogenomic predictions, natural compound- based drug repurposing represents a promising avenue for therapeutic intervention in liver cancer. Recent computational studies have demonstrated strategies to systematically identify bioactive natural compounds with potential efficacy against specific molecular targets, including GLS [50]. While our current study focused on pharmacogenomic correlations to prioritize candidate drugs, incorporating natural compounds could further expand the translational relevance of our findings and provide additional opportunities for developing targeted therapies guided by computational approaches. We acknowledge that certain clinicopathological factors, such as cirrhosis grade and systemic comorbidities, could influence GLS expression and therapeutic response. However, these details were not consistently available in the TCGA-LIHC dataset, which formed the basis of our analysis. Future studies with access to more comprehensive clinical data will be important to validate and extend our findings and to better understand how these factors may modulate GLS-related prognostic and therapeutic associations. We recognize that integrating structural modeling, inhibitor database information (e.g., ChEMBL, BindingDB), and drug sensitivity data from resources such as DepMap could provide more concrete evidence of GLS druggability. However, these analyses were beyond the scope of the current study, which focused on methylation-driven prognostic modeling and the clinical and immunological relevance of GLS. Future work will aim to incorporate these resources to strengthen the mechanistic and therapeutic rationale for targeting GLS in liver cancer.

Nevertheless, certain limitations should be acknowledged. This study mainly relied on bioinformatic analysis and preliminary organoid validation, and no direct mechanistic experiments, such as in vitro knockdown or in vivo functional assays, were performed to confirm the causal role of GLS in mediating drug sensitivity or immune microenvironment remodeling. The relatively small number of organoid samples was due to the need for voluntary patient participation and the lengthy culture process, which limited further stratified analyses based on clinical characteristics such as AFP level or vascular invasion. Therefore, the current findings should be interpreted as exploratory. Future studies will expand the sample size and incorporate CRISPR/Cas9 or siRNA-mediated functional experiments, as well as animal models, to validate the mechanistic role of GLS and its regulatory impact on the tumor microenvironment and therapeutic response. We also plan to integrate interpretable AI approaches such as SHAP or LIME to quantitatively assess feature contributions and enhance model transparency, especially with the availability of larger multi-omics datasets. Moreover, this study primarily focused on the established immune checkpoints PDCD1 and CTLA4 to evaluate the immunological relevance of GLS, while acknowledging that emerging targets such as LAG3, TIGIT, and VISTA may also play important roles. In addition, we recognize that immune molecular classification frameworks and the TCGA Pan-Cancer Atlas could provide valuable context for interpreting GLS expression, which will be incorporated in future studies to further elucidate its role within the broader immune landscape.

## 5. Conclusions

This study conducted a comprehensive bioinformatics analysis to identify methylation-driven genes associated with the progression of liver hepatocellular carcinoma (LIHC). Through this analysis, GNA14, GLS, and GNG4 were identified as key genes significantly linked to patient prognosis. A prognostic risk score model incorporating these three genes was constructed, demonstrating strong predictive performance for survival outcomes in LIHC patients. Among these, GLS emerged as a core gene, with its methylation and expression profiles providing valuable insights into tumor behavior and patient prognosis. GLS also showed promise as a biomarker for predicting sensitivity to immunosuppressants and chemotherapeutic agents, underscoring its dual role in prognosis and therapeutic guidance. These findings highlight the potential of GLS as a target for developing personalized treatment strategies, offering new avenues for improving clinical outcomes in LIHC patients.

### Clinical significance

The study developed a three-gene MDG-based prognostic model (GNA14, GLS, GNG4) with strong predictive power for 1-, 3-, and 5-year survival in LIHC patients. Among these, GLS was identified as a key biomarker associated with poor prognosis, emphasizing its potential role in risk stratification. A multivariate model incorporating clinical factors demonstrated moderate prognostic discrimination, and the inclusion of GLS methylation status further improved risk assessment. Notably, GLS methylation provided insights into the tumor immune microenvironment and showed potential as a predictive biomarker for immune checkpoint inhibitor (ICI) therapy. Additionally, its association with differential sensitivity to chemotherapy suggests a role in guiding personalized treatment strategies.

## Supporting information

S1 File Flowchart. (TIF)
