# Supplementary figures and images for: DNA methylation-driven gene-based drug response prediction model for liver cancer: The critical role of GLS
Source: PLoS One. 2025 Dec 5;20(12):e0338091. doi: 10.1371/journal.pone.0338091 (PMC12680199; doi:10.1371/journal.pone.0338091)

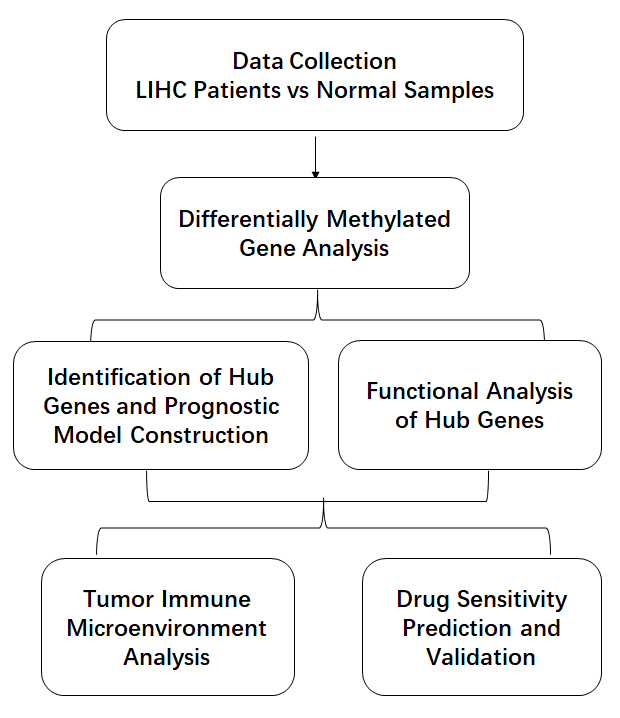

Supplement: S1 File — (TIF) [file pone.0338091.s001.tif]
